# The Third Man: comparative analysis of a science autobiography and a cinema classic as windows into post-war life sciences research

**DOI:** 10.1007/s40656-015-0080-z

**Published:** 2015-07-24

**Authors:** Hub Zwart

**Affiliations:** Department of Philosophy and Science Studies; Faculty of Science; Centre for Society and the Life Sciences (CSG); Institute for Science, Innovation and Society (ISIS), Radboud University, P.O. Box 9010, 6500 GL Nijmegen, The Netherlands

**Keywords:** Science autobiography, Philosophy of science, Psychoanalysis, Science and cinema, Lacanian psychoanalysis, Science ethics

## Abstract

In 2003, biophysicist and Nobel Laureate Maurice Wilkins published his autobiography entitled The Third Man. In the preface, he diffidently points out that the title (which presents him as the ‘third’ man credited with the co-discovery of the structure of DNA, besides Watson and Crick) was chosen by his publisher, as a reference to the famous 1949 movie no doubt, featuring Orson Welles in his classical role as penicillin racketeer Harry Lime. In this paper I intend to show that there is much more to this title than merely its familiar ring. If subjected to a (psychoanalytically inspired) comparative analysis, multiple correspondences between movie and memoirs can be brought to the fore. Taken together, these documents shed an intriguing light on the vicissitudes of budding life sciences research during the post-war era. I will focus my comparative analysis on issues still relevant today, such as dual use, the handling of sensitive scientific information (in a moral setting defined by the tension between collaboration and competition) and, finally, on the interwovenness of science and warfare (i.e. the ‘militarisation’ of research and the relationship between beauty and destruction). Thus, I will explain how science autobiographies on the one hand and genres of the imagination (such as novels and movies) on the other may deepen our comprehension of tensions and dilemmas of life sciences research then and now. For that reason, science autobiographies can provide valuable input (case material) for teaching philosophy and history of science to science students.

## Introduction

*The Third Man*, released on September 2, 1949, is regarded by many as one of the greatest cinema classics of all time.^1^ It was directed by Carol Reed (1949) and based on a screenplay by Graham Greene (who published a novel version in 1950), while the cast involved celluloid celebrities such as Alida Valli, Trevor Howard and Orson Welles. The movie is set in heavily-bombed post-war Vienna and tells the story of a racketeer (Harry Lime, played by Orson Welles) who makes money by clandestine sales of diluted penicillin, but manages to keep out of the hands of the military police (i.e., the four allied occupying powers) by travelling via the underground urban sewage system and, when things get rough, by simulating a fatal car accident and organising his own mock funeral. The novel begins with the arrival of a boyhood friend (Holly Martins), author of cheap Westerns, who adores Harry and comes to look for him (because the latter allegedly promised him a job), but is informed (upon arrival) that his hero has tragically died.

More than half a century later, in 2003, the autobiography of Maurice Wilkins (1916–2004) was published (shortly before his death), entitled *The Third Man of the Double Helix* (Wilkins 2003/2005). Wilkens, a world-famous biophysicist, had been awarded the Nobel Prize (in 1962) as co-discoverer of the structure of DNA (in 1953), together with James Watson and Francis Crick. At first glance, the connection between the movie on the one hand and the science autobiography on the other seems rather superficial. In fact, in the preface, Wilkins admits that he did not like the title to begin with:

The title of this book, *The Third Man of the Double Helix*, is not the one I would have chosen. I have deferred to the advice of my publishers on that issue! (p. x)

In other words, the book begins with a disclaimer: with an author discarding the title of his own book as arbitrary and external, apparently selected because of its catchy familiarity; because of the film no doubt,^2^ something of an embarrassment to a serious academic like Wilkins.

Yet, as I will argue in this paper, there is much more to the book’s title than its familiar ring. First of all, both events (the making of the movie and the complicated process which led up to the discovery of DNA) coincide in time; they belong to one and the same period: the post-war years, an era during which academics, not in the least Nobel Laureates, and notably physicists (such as Wilkins, who had joined the Manhattan project during the war) tended to reflect quite extensively on their societal role as scientists. Had their discoveries indeed conferred ‘benefit on mankind’, as it is so elegantly phrased in Alfred Nobel’s Will?^3^ Now that Evil (in the form of the Axis Powers) had been defeated, the ‘other side’ of the cataclysmic drama known as World War II (exemplified by the ruins and rubbles of demolished cities in Germany, Austria and Japan) came into full view, presenting a grim foreboding of the scale of destruction that would occur should a Third (nuclear) World War become reality.^4^

Moreover, to the extent that the book’s title was indeed an embarrassment to its author, this only makes it all the more interesting because, as we will see, a whole series of ‘embarrassments’ are recorded in Wilkin’s memoirs. Indeed, one could argue that ‘embarrassment’ functions as the book’s typical mood, something comparable to nausea in the case of Sartre, boredom in the case of Heidegger and discontent in the case of Freud. Most if not all of the scientific highlights recorded in Wilkin’s autobiography are accompanied by instances of (at times profound) embarrassment. And Wilkins himself admits as much when he quickly adds (right after the disclaimer quoted above) that the title “does resonate with some of the tensions, accusations, confusions and controversies that have attended the telling and retelling of the DNA story” (p. x).

In fact, the more we dive into the movie (and the novel) on the one hand and Wilkins’s scientific memoirs on the other, the more their congruencies begin to multiply. In other words, although at first glance (besides the obvious fact that Wilkins was indeed the ‘third man’ to be credited with the discovery of DNA, next to Watson and Crick), any further connections seem unlikely, on closer inspection, a detailed ‘comparative analysis’ of the documents reveals that their family likeness is actually quite significant. In many ways, they address similar themes, conflicts, ambivalences and hopes, so that the movie/novel elucidates some of the key motifs in the autobiography and vice versa. Taken together, these documents provide a window into the socio-cultural ambiance of post-war cutting-edge molecular life sciences research, focussing on DNA.

But before turning to the details of my analysis, let me first address the question why I find it relevant to study science autobiographies such as Wilkins’s memoirs in the first place and, subsequently, why comparisons with movies (or other genres of the imagination, such as novels or drama) can be helpful for our purposes.

## Why science autobiographies?

Science autobiography is a genre of writing with a long history. Many scientists, notably prominent ones (‘aristocrats of science’) have written memoirs, from concise fragments up to voluminous monographs (albeit not always explicitly intended for publication). But although autobiographical sources often contain a wealth of data concerning science as a ‘practice’ (science *in action*), in science studies as a research field (notably during the past decades) autobiographical sources tended to be regarded with suspicion. And this has been the case, one could argue, for two reasons: on the one hand because they are *auto*-biographical and on the other hand because they are auto-*biographical*.

First of all, as ego-documents, written by the individuals themselves, they often prove unreliable, either because of the fallibility of human memory, or because authors of science autobiographies often have very specific self-serving objectives in mind, a strategic ‘agenda’ so to speak, such as: disproving certain accusations of tinkering and fraud, or challenging accounts published by others which they (for whatever reason) consider biased or damaging to themselves. Thus, science autobiographies can hardly be regarded as impartial. But the wide-spread reluctance to making use of autobiographical sources in science research goes much further and is actually fuelled by scepticism towards biographical (i.e., individual-oriented) approaches *as such*. As Söderqvist (2007) has argued, notably during the 1970s and 1980s, the large majority of post-war historians and sociologists of science tended to be overtly “anti-biographical” (p. 252). Indeed, as Söderqvist phrases it, no genre of history fell under more “odium” in academic science research than that of science biography, prominently present in the public domain, but hardly visible in scholarly discourse. As a rule, biographical sources were “despised and dejected” by academics, while pro-biographical pleas for close studies of outstanding individuals, such as Thomas Hankins’s paper “In defence of biography” (1979), formed the exception to the rule.

One important causal factor was that scientific ‘life writing’ had become associated with the ‘great men’ or ‘heroes of science’ approach, discredited for various (notably methodological) reasons. Especially during the 1970s and 1980s, science studies as a research field opted for a sociological rather than a psychological (let alone introspective) stance. Scientific research was no longer seen as the work of individuals, but rather of networks, even of ‘actor networks’ in the Latourian sense (including both human and non-human ‘actants’). Thus, the “dismissal” of biography as a credible and usable source seemed an inevitable methodological implication of the broader sociological dismissal of the individual as a legitimate focus of research in science studies as such (Shortland and Yeo 1996). Building on Barthes (1968), Foucault (1969/1995) had proclaimed the ‘death’ of the individual scientific author (as an autonomous creator of insight and truth). By default, scientific research was regarded as a collective practice, a ‘discourse’ in the Foucauldian sense, and as the work of more or less anonymous individuals, whose names were published and listed for technical purposes (such as: retrieval of information, or performance assessments of research teams).

This is already implied in the methodology of science itself, of course, which basically consists in the systematic eradication of subjectivity, of “pathological experiential reality” (Žižek 2013, p. 134; where ‘pathological’ is used in the Kantian sense of the term). Ideally, the scientist as an individual should be replaceable, either by other trained individuals or by automation. This waning of the individual (this cleansing of science of all-too-personal dimensions) encouraged a downgrading of individual-oriented approaches, although there are notable exceptions to this rule, such as Evelyn Fox Keller’s famous biography of Nobel laureate Barbara McClintock (Keller 1983), focussing on gender dimensions and the role of women in science, consciously counterpointing the erstwhile masculine ‘heroes of science’ tradition mentioned above (cf. Govoni and Franceschi 2014).

In recent years, however, interest in science biographies among science studies scholars has revived (Shortland and Yeo 1996). Söderqvist (2007) refers to this revitalised interest (since the 1990s) as the “renaissance” of biographical writing. Not in the sense that scientific breakthroughs are now once again regarded as the unique achievements of solitary scientific ‘heroes’, but rather because it is now more readily acknowledged that science autobiographies may contain detailed and valuable (“high resolution”) information concerning the “micro-level” of scientific research (Zwart 2008a): the context of discovery, the processes of life-long learning, the various shifts in technology and methodology scientists have to adapt to, as well as the intricate real-life moral dilemmas they encounter and, last but not least, the interwovenness of scientific research as a practice with other domains of culture, such as politics or art. Autobiographies provide us with a “view from inside”, as it were, presenting critical assessments by scientists themselves of their own performance and roles, both in the academic and in the public sphere, notably during moments of controversy or crisis (van Rijswoud 2010, p. 146).

In fact, science autobiography has become a prolific genre. Since the publication by James Watson of *The Double Helix: a Personal Account of the Discovery of the Structure of DNA* in 1968 (the first classical example of a contemporary science autobiography), various scientific breakthroughs (from the discovery of the DNA structure in 1953 up to the Human Genome Project in the 1990s) have given rise to a plethora of science autobiographies, as a ‘by-product’ of life sciences knowledge production (Zwart 2008a). In terms of style, tonality and genre conventions, science autobiographies are very unlike other textual products of scientific inquiry. Whereas (as pointed out above) normal research papers are characterised by a grammar of ‘anonymisation’ (a tendency to erase or repress any idiosyncrasies of the researcher-as-an-individual in terms of style and vocabulary), in science autobiographies the individual Self emphatically takes the floor. Therefore, in terms of atmosphere and structure, science autobiographies are often much more similar to novels, plays or movies than to the more standardised forms of scientific writing. And whereas normal research papers tend to focus solely on the science, science autobiographies rather reveal the extent to which research practices are pervaded (‘infected’) by the dynamics of the socio-cultural world, for instance in the form of political convictions, intimate relationships, psychic problems, fierce competition and personal ambitions. They not only tell us something about science as such, but also function as documents reflecting the Zeitgeist of a particular era. And this is one important reason why a systematic comparison between science autobiographies and other cultural genres, such as novels and movies, can be revealing. Science autobiographies effectively bridge the gap between what are often presented as separate, compartmentalised ‘cultures’, such as science and politics, science and religion, or science and art.

Up to a certain point, the relevance and value of science *auto*-biographies may be seen as comparable to that of ‘*hetero*-biographies’ (i.e., scientific life writing by *others*). Rather than presenting science as a linear progress, both auto- and hetero-biographical sources inform us about flashes of intuition, lucky breaks, missed opportunities and fatal turns (Beckwith 2002; Graham 2004). Söderqvist (2006, 2011) distinguishes seven possible uses of scientific biographies (seven answers to the question why is makes sense for science scholars to become interested in scientific life writing), namely: (1) as a method for writing *contextual* history; (2) as a means for understanding science-in-the-making; (3) as an effort to promote popular understanding of science; (4) as belles-lettres; (5) as public commemoration (eulogy); (6) as a labour of love; and finally (7) as an exercise in research ethics. With the exception of (5) and (6), these answers apply to autobiographies as well. In this paper, however, I will notably focus on (1) and (7). First of all, autobiographies tend to portray scientists as worldly beings whose laboratory practices resonate with evolving socio-cultural tendencies in the world at large. Thus, Wilkins’s memoirs will be used to *contextualise* post-war biophysics (culminating in the discovery of DNA) vis-a-vis contemporaneous developments in other socio-cultural domains. Furthermore, Wilkins’s memoirs provide us with intriguing source material for ethical reflection, not in the sense that the author is a role-model or ‘exemplar’ (along the lines of the scientific hero-approach), nor in order to expose a famous scientist as a misconducting, plagiarising fraud (the ‘debunking’ approach to life writing), but rather in the sense that his memoirs have something important to say (in a detailed, lively manner) about the moral dilemmas scientists come to face, as well as about the strategies they develop for dealing with them. In other words, in autobiographies, laboratory life emerges as a practice of the self, as an instance of ‘individuation’, while the process of writing memoirs *itself* may be regarded as a crucial component of the individuation process.

A final reason for becoming interested in science autobiographies is that, for me as a continental philosopher of science, psychoanalysis is an important source of inspiration, a key component of my conceptual frame of reference. And seen from a psychoanalytical perspective, science autobiographies present ‘case studies’, not in the traditional ‘pathographical’ sense of the term (with scientists featuring as patients tormented by various neurotic symptoms), but rather in the sense that these self-portrayals give voice to the fascinations, inspirations, flaws, obstacles and challenges of researchers working in a particular socio-cultural ambiance,—provided these documents are read in a particular way and from a particular angle. In this paper, Wilkins’s memoirs will be analysed from an ‘oblique’ perspective, in the sense that the focus will not be on the scientific details as such (on luminescent crystals and deoxyribonucleic acid, Wilkins’s favourite research topics), but rather on the existential tensions and conflicts of Wilkins as a successful but at the same time tormented researcher (the ‘micro’ level), as well as on the various connections between scientific problems and broader socio-cultural challenges (the ‘macro’ level).

It is against this backdrop that a systematic comparison with *The Third Man* as a cinematic classic can prove revealing. The doubts, controversies and challenges of Wilkins as a prominent scientist—meticulously articulated in his (highly personal) autobiographical account—in many ways reflect the broader conflicts, doubts and challenges of a whole era: the post-war context, as captured by the classic movie. For although the autobiography purports to be ‘realistic’, while the movie purports to be ‘fictional’, both sources provide a window into a particular socio-cultural environment: post-war Europe during the late 1940s and early 1950s (from the end of World War II up to 1953, the year of the DNA discovery, but also of Stalin’s death).

My comparative analysis involves a number of successive steps. First, I will focus on the connection between science and warfare (between science and the death drive so to speak), especially during the post-war years. After pointing out important similarities in terms of ambiance (between Lime’s Vienna and Wilkins’s London), I will zoom in on two congruous scientific developments that played a significant role in the allied war effort, although initially they may seem unconnected: on the one hand quantum physics and the Manhattan Project, on the other hand the discovery and large-scale production of penicillin. Whereas in the first case the darker, demonic aspect of scientific progress seems obvious, the second development had its collateral damage as well, notably in the form of black markets and clandestine sales. In both cases, the societal profile of science proved fairly ambiguous. Subsequently, attention will shift from the (alluring but treacherous) ‘object’ of research (nuclear energy, antibiotics, DNA) to the tormented ‘subject’ of research: the scientific researcher who is challenged by moral dilemmas, notably concerning the handling of sensitive information. I will re-assess the famous controversy over the question whether the use by Wilkins, Watson and Crick of ‘photograph 51’, belonging to Rosalind Franklin, was morally objectionable or defendable. Finally, attention will be given to the relationship between science and art, or more precisely: to the interconnectedness between beauty and destruction, as addressed by both Wilkins and Lime.

## The post-war years: the mutual entanglement of science and warfare

One of the most interesting dimensions of Wilkins’s book is the light it sheds on the mutual entanglement of modern science and modern warfare. On the broader socio-historical level, this entanglement has already been documented by scholars such as Kay (2000), pointing out the intimate connections between DNA research and nuclear bombs, between molecular biology and the Cold War. Indeed, Kay meticulously explains how, during the second half of the Twentieth Century, life sciences research at top ranking universities became thoroughly “militarised”.

This interwovenness of science, politics and the military is clearly reflected in Wilkins’s memoirs. Throughout his life, Wilkins was an ‘engaged’ scientist in a rather outspoken way, quite conscious of what he refers to as the “political element in science” (Wilkins 2003/2005, p. 35). During his student days at Cambridge, he became a member of the Communist Party (from which he resigned in 1939 because of the Hitler-Stalin Pact), meanwhile experimenting with home-made bombs as a preparatory exercise for joining the resistance, should Germany’s plans for invading the United Kingdom prove successful. As a budding academic researcher, he became involved in studying luminescent crystals, from which wartime radar technology emerged. Indeed, as Wilkins argues, the beams of microwaves developed by physicists like himself (able to penetrate the foggy atmosphere often covering the British Isles) significantly contributed to Allied air supremacy, a decisive factor in the War effort, notably during the Battle of Britain. Later on, he went to Berkeley to join the Manhattan Project, to help prevent Germany from winning the nuclear arms race, but he was struck with horror when he learned (via the newspapers) in August 1945 that two Japanese Cities had been destroyed, using the technology he himself had helped to bring about. Thus, he was confronted with the Janus-head of science, unleashing both salvation and destruction.

Right after the War, prompted by these dramatic experiences, he began to look for “more positive applications of my skills” (p. viii). He decided to shift his focus of research from ‘luminescent’ to ‘aperiodic’ crystals (i.e., genes) and from *nuclear* physics (the science of death) to *bio*-physics (the science of life), bringing with him his physical technologies (such as crystallography) and his mathematical tools (such as Bessel function calculation). Wilkins clearly hoped that molecular biology (initiated by physicists such as Max Delbrück and Erwin Schrödinger) would prove more life-friendly and humane than the Manhattan Project, whose horrific outcome had nearly driven him out of science (2003/2005, p. 88). Yet, the life sciences, where he and so many other physicists sought refuge during the aftermath of the War, soon revealed their own Janus-face aspect as well. Insights and products derived from life sciences knowledge may likewise be used for disruptive rather than beneficial purposes, for instance in the context of biological warfare. Knowledge and know-how may fall into the wrong hands,—an issue which is nowadays often captured under the heading of ‘dual use’.

Similar dilemmas are mirrored by the movie. In the following, I will briefly focus on three aspects. First of all the unprecedented destructive power of wartime bombardments (culminating in the use of atomic bombs). Secondly, the connection (less obvious perhaps) between penicillin and World War II. And finally, the handling by scientists of sensitive information, notably in the context of espionage and competition. In all three cases, although the contexts of movie and memoirs may seem different, a mutually revealing ‘family likeness’ can be discerned between them in terms of basic ambiance.

## Bombed about a bit

Post-war Vienna was a severely demolished urban area (albeit by Allied air raids and Soviet shelling rather than by atomic bombing). A former metropolis has been effectively transformed into dreary heaps of “undignified ruins” (Greene 1950/1977, p. 14), almost beyond recognition (“bombed about a bit”, as the movie phrases it). After the bomb raids came the occupation, by the four allied powers (the Soviet Union, the Unites States, the United Kingdom and France), who divided the city into occupational zones, while the *Innenstadt* was policed by international patrols. Each patrol team consisted of four members (one for each of the four powers) who as a rule were neither able to communicate among themselves nor with the Viennese populace, so that bizarre linguistic and cultural confusions unfolded on a daily basis. All this resulted in a most surrealistic and outlandish “lawscape” (Philippopoulos-Mihalopoulos 2014), captured quite convincingly by the movie.

German and Austrian cities were not the only European cities heavily scarred by air raids of course. As Wilkins describes in his memoirs, cities like London and Birmingham had had their share of bombardments as well. While the black-and-white movie presents the dead and gloomy streets of post-war Vienna, Wilkins’s memoirs describe the damage inflicted on British urban centres. In fact, the physics laboratory at King’s College, where he did most of the research that contributed to the discovery of the structure of DNA, was built in a bomb crater (“The College authorities agreed to use a big bomb crater in the College quadrangle to house new labs for the influx of researchers. So Randall’s lab grew out of a bomb crater”, Wilkins 2003/2005, p. 99).

In the movie, some of the most dramatic scenes are situated underground: the sewage system, Vienna’s *Acheron* as it were, described by Greene as strange unknown world, a cavernous land of waterfalls and rushing rivers. But there is a similar underground dimension to Wilkin’s memoirs as well:

The chemistry department had a big [X-ray tube] set. It was deep in their basement below the Thames, and to get there we had to walk through long passages in the dark. But before going down the stairs we passed a window looking across the Thames, where I liked to catch a glimpse of a big illuminated sign that spelled out ‘OXO’ in bright lights on a high tower. I liked the OXO sign … the big X in OXO presaged the famous X pattern that would appear in our X-ray photographs of DNA (p. 125)

These similarities in ‘physiognomy’ of post-war urban areas, certainly add to the congruence in atmosphere and ambiance between the movie and crucial chapters of the memoirs, recounting the discovery referred to in the title. Yet, as I will argue, this congruence not only pertains to the dramatic backdrop as such, but also to the dramatic events that are enacted within this setting. Let us therefore now shift attention from backdrop to action.

## Nuclear bombs, penicillin and DNA: a common denominator?

As indicated, whereas *The Third Man* as an autobiography focuses on the vicissitudes of a British physicist leaving the field of quantum physics to study DNA, *The Third Man* as a movie tells the story of a penicillin racket set in post-war Vienna. At first sight, the connection between these themes seems minimal. Could any two ‘products’ of modern scientific research be further removed from each other than (life-saving) penicillin and (life-threatening) atomic bombs? And yet, again, on further reflection it turns out that both products convey quite similar moral ambiguities.

To begin with, a number of ‘synchronicities’ can be discerned between the production of penicillin (a key theme in the movie) and the making of the atomic bomb (a key endeavour in Wilkins’s memoirs). Penicillin was discovered in the 1920s, the ‘golden age’ of quantum physics (the research field from which the nuclear bomb evolved). Alexander Fleming, who is formally credited with the discovery of penicillin, dates his (accidental) finding on Friday morning, September 28, 1928 (1 year after the famous fifth Solvay conference on protons and electrons). Still, the transition of penicillin from a laboratory entity into a marketable pharmaceutical product, producible on a massive scale, proved a daunting task. Penicillin was made available for mass production by Merck in 1942, but the War effort significantly accelerated the process. In July 1943, the U.S. War Production Board drew up a plan for the mass distribution of penicillin to Allied troops fighting in Europe, to ensure supremacy of Western armies over enemy forces. For the latter, antibiotics were not available. Thus, the turn towards massive production of penicillin, as part of the military ‘equipment’, coincided in time with (and was directed towards achieving similar goals as) the Manhattan Project (the application of nuclear physics on a massive scale).

These aspects (the initial scarcity of penicillin and the fact that it entered the market first and foremost as a ‘militarised’ product) are also reflected in Wilkin’s memoirs. During childhood, or instance, his sister suffered from septicaemia and barely survived a series of debilitating operations because “there were no antibiotics then” (p. 17). And during the War, Wilkins himself met with a similar fate: he contracted a severe ear infection and spent 7 weeks in a hospital bed in Berkeley, having holes drilled in his head, because penicillin, although technically available, was not yet producible on a large-enough scale (“no penicillin for me, only for the fighting men”, p. 80). In other words, both movie and memoirs confirm the vital strategic significance at that time of a medicine whose availability is nowadays taken for granted.

The movie focusses on living conditions in devastated Vienna during the aftermath of the War, and as such, contemporary reviewers considered it a fairly accurate and convincing visual and emotional portrayal (Lynette 1978). Hunger, poverty and infectious diseases were ubiquitous, together with bitterness, cynicism, and distrust, fear, opportunism and despair. And penicillin was first and foremost a *military* product, in the hands of the occupational powers. Under such circumstances, given the power imbalance between occupants and denizens, and the virtual absence of civil society and democratic institutions, a black market for pharmaceuticals, based on plundering army provisions, was likely to evolve.^5^ As Greene explains in the *Preface* to the novel (1950/1977), the story of the penicillin racket was based on grim truth, involving members of the Royal Air Force selling penicillin and other medicinal products in poverty-stricken Vienna (cf. Wapshott 1994, p. 200). Moreover, clandestine practices such as dilution (to increase profit) could easily turn the medicine in question into poison—remember that, etymologically speaking, the word φάρμακον means both medicine and poison in ancient Greek, and the same applies to ‘gift’ (‘present’ or ‘talent’ in English) and ‘Gift’ (‘poison’ in German). Thus, although penicillin was originally developed as a benefit to humankind, substantial ‘collateral damage’ could be involved, in connection with post-war political circumstances.

No clear moral ‘message’ is conveyed by the movie and it consistently refuses to take position on political issues (Beer 2001), focussing instead on human drama and living conditions under post-war circumstances.^6^ Still, it is pointed out how, as a by-product of the liberation, the Allies introduced bio-medical pollution into post-war Austria. And although Harry Lime was a doctor rather than a scientist, the penicillin racket he set up indicates that Wilkins’s escape into the safe haven of the ‘sciences of life’ did not put an end to doubts concerning the ‘benefits’ conferred by twentieth century science on humankind. Even a life-saving medicine such as penicillin could become a life-threatening evil when misused and tainted by ‘wrong’ hands.

What goes for penicillin goes for nuclear information as well: both were very much sought after in those years. Both entities were things of tremendous value. Their availability or absence represented a decisive factor in matters of life and death, or even victory and defeat. As targets of attention, however, they proved difficult to control. Both nuclear information and penicillin were crucial ingredients of the war effort, and both emitted a tempting appeal: STEAL ME, albeit in the one case notably because of political persuasions, in the second case predominantly for pecuniary motives. What goes for uranium or plutonium also applies to penicillin. Such entities may constitute what Lacan refers to as the ‘object *a*’ of the research fields in question, of high energy physics and biomedical research respectively: life-saving and alluring, but at the same time dangerous, elusive and impossible to contain. In the case of nuclear bombs, the goal is deterrence, in the case of penicillin, immunity, but the envisioned effects may be lost in the longer run, due to proliferation (of atomic weapons) or increased resistance (of bacterial strains to antibiotics). This (in addition to the short-term damage) is what made nuclear espionage and antibiotics rackets so damaging. Although spies often worked for a cause, while Harry Lime worked for a profit, their impact was by and large the same: they contributed to the proliferation of a (potentially dangerous) scientific product of significant value which only achieves its goals when it is exclusively used by the ‘right’ hands.

Furthermore, a number of family likenesses can be discerned between penicillin and DNA as well. For both entities, purity is a key issue. From the scientific point of view, both entities must be as pure and clean as they can possibly get. A significant portion of Wilkins’s memoirs is dedicated to his efforts to produce pure, undiluted strands of DNA: ‘purified’ DNA, almost ‘pristine’, up to perfection. At a certain point, for instance, he tells us that his DNA was so “excellent” and “pristine” that is was shouting at him, “Look how regular I am!” (p. 124).^7^ Impurity is evil. Like pure DNA, high quality penicillin is a costly and profitable product, but diluted versions can prove deadly poison, while impure DNA leads researchers in wrong directions, causing them to err concerning its basic structure.

In this section, the focus has been on the role of the alluring, action-provoking object (from radioactive elements via antibiotics up to DNA). In the next section I will argue that, besides backdrop and object, the congruence between movie and memoirs pertains to the subject-position as well. In both documents, key protagonists are confronted with similar (and therefore mutually revealing) moral ambiguities and dilemmas.

## Handling sensitive information

Another by-product (accompanying activity) of warfare is espionage: a second intriguing motif in Wilkins’s memoirs. During his left-wing pre-war years, but also as a prominent scientist, he befriended, or at least became acquainted with, a number of individuals who were actually recruited to spy for the Soviet Union, or even convicted for espionage during the Cold War period. Several Cambridge graduates, who, like Wilkins, had been members of the Communist Party, were accused of giving classified information to the Soviet Union. One of the convicted spies whom Wilkins befriended was Klaus Fuchs (1911–1988), who had joined the Communist Party in Germany, but fled to the United Kingdom in the 1930s where he became enrolled in the British atomic bomb project, moving over to the Theoretical Physics Division at Los Alamos in 1944. He passed information on these projects to the Russians via a spy codenamed ‘Sonia’.^8^ In his memoirs, Wilkins introduces Fuchs by saying that he (Fuchs) seldom said anything and that he (Wilkins) was very surprised to learn that he was in fact a spy (2003/2005, p. 79).

Another famous spy who figures in Wilkins’s book is Allan Nunn May. Like Wilkins, he studied in Cambridge, joined the Communist Party in the 1930s and became involved in radar research during the early War years. At a certain point it was discovered that, on various occasions, he had passed confidential information to the USSR. Apparently, the police waited outside the door of a lecture theatre where Nunn May was teaching and arrested him as he came out (p. 97). Due to the sudden arrest of the person who at that time was considered the most likely candidate to be appointed as Chair in Physics at King’s College London, John Randall was suddenly offered the position, who in his turn appointed Maurice Wilkins as his deputy. Had Nunn May not been arrested, Randall’s career at King’s would never have happened, Wilkins would not have been invited to London, and the discovery of DNA might have taken a completely different turn. Another spy whom Wilkins befriended during his Cambridge days was Arthur Hone, who disappeared into the Soviet Union. When Wilkins later visited the USSR himself, he failed to retrieve any information concerning Hone’s whereabouts (p. 262).

Again, at first glance, the congruence between movie and memoirs seems limited. Harry Lime is involved in clandestine penicillin sale rather than espionage. And yet, the movie’s conspiratorial atmosphere of secrecy and cover-ups, of suspicions and arrests is reminiscent of the autobiography in several ways. To begin with, we must keep in mind that during the War, Graham Greene himself had been a secret agent working for the British Intelligence, a close colleague of Kim Philby, the archetypal double agent, one of Britain’s most famous spies, and a prominent member of the notorious Cambridge spy ring (Sheldon 1994). Philby and Greene met in 1943 in Spain and became close friends. Greene wrote an introduction for Philby’s memoirs *My Silent War*, written in Moscow in 1967 and published in 1968. Philby was exposed as a double agent in 1963, much later than two other co-members of the Cambridge ring, Guy Burgess and Donald Maclean, who had already defected to the Soviet Union in 1951. But because of his close association with Burgess and Maclean, Philby became a suspect as well. He was interrogated by the secret service (MI5) and actually dubbed the “third man” (Drazin 1999, p. 144; Brown 2014, p. 30). In other words, besides Lime (as the third man of the movie) and Wilkins (as the third man of the DNA story), Philby is a ‘third’ third man, and many have argued that Lime was actually modelled on Philby. And indeed, there are some intriguing similarities to point out, not only between Philby and Lime, but even between Philby and Wilkins. Let me address these in more detail.

The view that Philby functioned at least partially as a model for Harry Lime is broadly accepted (Drazin 1999; Beer 2001). Philby went to Vienna in February 1934 during the Austrian civil war, joined the besieged socialists and actually helped many of them to escape through the sewers. In June 1934, he was recruited by a Soviet agent. Subsequently, he met and probably recruited Hans Peter Smolka. But he also met a young communist named Litzi Friedmann with whom he fell in love and whom he provided with a British passport by marrying her. In the movie, Harry Lime likewise provides his girlfriend Anna (daughter of a Czech Nazi hiding out in Vienna under a false name) with a counterfeit passport. Hans Peter Smolka went to London, where he changed his surname into Smollett, but returned to Vienna after the War. Here, he met Graham Greene in February 1948, who had come to Vienna to do research in preparation of *The Third Man* (Drazin 1999). Quite probably, the two men already knew one another, via Philby. Smollett, who acted as a kind of intermediate (‘third’) man between Philby and Greene, had written Viennese stories about escapes through the sewers and diluted antibiotics, which he handed over to Greene, hoping that the latter would help him find a publisher, but Greene decided to build some of Smollett’s motifs into his own screenplay instead.

As a result, there are some striking parallels between Philby and Lime: from the sewers escapes up to passports serving as retribution for love. Given these various parallels between Lime (and Martins) on the one hand and Philby (and Greene) on the other, *The Third Man* has been regarded as Greene’s effort to exonerate himself from his embarrassing friendship with a double agent of world-renown. Much like Holly Martins, Greene must have been torn between his personal affection for Philby (with whom he had been quite close) and the latter’s misdemeanours: not only his defection to the Soviet Union, but also the ease with which he organised (or stoically accepted) the elimination of numerous individuals (including friends, colleagues and lovers) who somehow stood in the way or had become a risk factor. In a similar way, Wilkins in his memoirs faces the task of clarifying his relationships with the spies he befriended.

It has become known, moreover, that, similar to Philby, Wilkins himself had actually been investigated by MI5 between 1951 and 1954 (Travis 2010; Gann and Witkowski 2012). He was suspected of having shared information from the Manhattan project with communist party members, who then transferred these secrets to the USSR.^9^ Anonymous sources (probably colleagues) informed MI5 that Wilkins, besides displaying “strong left leaning tendencies”, allegedly held the view that passing on information connected to atomic energy was justifiable since scientists were free to use their knowledge the way they wanted and atomic knowledge should be shared within the international scientific community. On the basis of a written report on him (provided by an anonymous informant, working with him at King’s), Sir Harold Himsworth, Secretary of the Medical Research Council, was asked to provide a formal assessment. Eventually, Wilkins was described as “a caricature of a scientist”, “incapable of dealing with ordinary human situations” and in consultation with psychoanalysts, and the case was dropped (Travis 2010).

Espionage was a hot topic during the early 1950s. In fact, 1953 was not only the year of the unravelling of DNA, but also the year in which Ian Fleming (a former naval intelligence officer) published *Casino Royale*, the first of his highly successful James Bond novels.^10^ As Drazin (1999, p. 151) notices, Vienna was one of the hotspots of the international espionage scene, so that the fact that spying is never explicitly mentioned in the film is somewhat surprising.^11^ But this can be regarded as ‘displacement’, in the sense that some key features of Philby (the double agent) were actually transferred to Harry Lime (the racketeer). For Wilkins himself, however, the key embarrassment, and the key motive for writing his memoirs, was not his relationships with spies, but rather the controversies that had arisen concerning his role in the DNA discovery. But here as well, like in espionage, the handling of sensitive scientific information was the core issue. The memoirs are structured as a kind of self-investigation: an effort to reconstruct the facts of an embarrassing episode.

Various similarities between movie and memoirs can be pointed out in this respect. As indicated, the movie tells the story of a visitor traveling to Vienna to look for an old friend. The accusation that Harry had diluted penicillin with coloured water (Greene 1950/1977, p. 80), causing young children to die from meningitis or become incurably debilitated, is initially discarded as utterly incredible. Apparently, Harry is being demonised by the police and it takes some time for his former friend to realise that there had always been this other, darker side to Harry, that he had always been something of a swindler. Step by step, the visitor is drawn into a new role: that of a (blundering, naive) investigator, realising and assuming his moral responsibility (Gomez 1974, p. 335).^12^

But precisely this same atmosphere of growing suspicion is at work in the autobiography as well. In his *Preface* Wilkins explains that he had written his memoirs because his contribution to a Nobel Prize-winning discovery had become tainted by controversy. Like in the case of espionage described above, he had become the target of “investigations” (by science scholars such as Sayre 1975) and “accusations” (by feminist “activists”, p. ix). He therefore felt challenged to present his own version of the events. Interestingly, like in the case of Cold War espionage, the dispute concerned the admissibility or inadmissibility of passing on sensitive scientific information to competitors in a race, albeit a scientific race rather than an arms race. Let us look at the details more closely.

## The purloined photograph

“Has it occurred to you … that you might dig up something—well, discreditable to Harry?”

(The Third Man, *novel version*)

Intellects of the vaster capacity, while more forcible, more constant, and more eventful in their movements than those of inferior grade, are yet the less readily moved, and more embarrassed and full of hesitation (*Edgar Allan Poe,* The Purloined Letter)

For Holly Martins, the author of cheap Westerns visiting Vienna, Harry Lime was a childhood hero, as we have seen. It took some time for him to accept that Harry was actually a fraud. Gradually, it dawned on him that there was something queer about his friend’s ‘tragic death’, that there was something wrong about the case, something “discreditable to Harry” (Green 1950/1977, p. 37). Eventually, a Gestalt switch occurred, and the one-time hero metamorphosed into a villain.

Interestingly, a somewhat similar scenario evolves in the case of the DNA discovery. At first glance, it is a story featuring three scientific heroes. Gradually, however, notably after the publication of Watson’s personal account *The Double Helix* in 1968, something seems to be the matter with this discovery (which had resulted in a Nobel Prize, one of the most famous Nobel Prizes ever awarded)—something potentially discreditable to Jim, and to Maurice, and (to a lesser extent, perhaps) to Francis.

As indicated, Wilkins already brings the embarrassing issue up in the *Preface* to his book. The quintessence of the story is that James Watson and Francis Crick partly based their ground-breaking discovery on a crucial piece of evidence: an X-ray diffraction photograph belonging to Rosalind Franklin (who tragically died of cancer before the Nobel Prize was awarded, in May 1952), known as ‘photograph 51’ and passed on to them (without Franklin’s explicit permission) by Wilkins.

To understand the significance of this event, we must cast a quick glance at the way in which the DNA structure was actually unravelled. In essence, the DNA discovery entailed two competing teams: the team at *King’s College*, which initially took the lead in the DNA race (and which, besides Wilkins, consisted of researchers such as Rosalind Franklin and Raymond Gosling) and the team at *Cambridge* (Watson and Crick). The Cambridge method basically consisted of model building, not only in the literal sense (piecing together bits of wire and metal to come up with a plausible three-dimensional structure), but also in the figurative sense: putting together pieces of information collected from others, such as Chargaff’s discovery concerning the 1:1 ratios of base-pares, an important clue for solving the DNA puzzle.^13^ Watson and Crick were not directly involved in ‘hands on’ research themselves. Officially, they worked on other topics (haemoglobin and bacteriophages). Their “gay” and carefree mental labour on DNA was not done in a laboratory, but during discussions and walks (Zwart 2013). Thus, they collected and connected crucial fragments, pieces of the puzzle, coming from various sources (Paulin, Chargaff, Franklin, etc.). Franklin unwittingly contributed a superb crystallographic (‘B pattern’) photograph,—‘*photograph 51*’, taken in 1952—, which she had been keeping to herself. Wilkins describes the controversial event in two different chapters of his memoirs (Chaps. 7 and 8):

The most disturbing question relates to an X-ray diffraction photograph that was … lying in a drawer in Rosalind’s office… This photograph was particularly clear, and everyone agreed subsequently that it provided important pro-helix evidence. I was given the pattern by Raymond [Gosling, Franklin’s collaborator] on 30 January 1953, when Rosalind was preparing to leave our lab. Why would she, despite having found this evidence, give us an account of why DNA was not helical? (p. 184)

Then, something extraordinary happened. One day in January 1953, Raymond met me in the corridor and handed me an excellent B pattern that Rosalind and he had taken… Raymond made it clear that I was to keep the photograph! The new photograph was almost as extraordinary as its being shown to me. It was much clearer and sharper than the first clear B pattern that Rosalind had shown us in October 1951… The new pattern showed the helix X-shape more clearly than ever before. Raymond gave me to understand that Rosalind was handing the pattern over to me to use as I wished.

A few days later Jim [Watson] was visiting us, and I stopped him in the main passage of our lab to show him the photograph. I said that it was very frustrating that Rosalind was continuing to base her work on non-helical ideas even though she had this new pattern that was even more convincingly helical than ever. As I stood with Jim in the corridor … I felt I must tell him what I had been thinking … [but] I got no further than saying ‘I think Chargaff’s ratios are the key to DNA structure’ … and Jim said ‘I do too’ before he hurried off. (p. 197/198)

In retrospect, all key sources—autobiographical accounts by Watson (1968/1996), Crick (1988) and Wilkins (2003/2005) as well as biographical accounts concerning Franklin (such as Maddox 2002)—agree that these two corridor events, revolving around an allegedly ‘purloined photograph’, constitute a turning point in the story of the discovery of the structure of DNA.

The embarrassment stems from the fact that an element of theft or academic ‘espionage’ seems involved in the DNA story and several critics have argued that Wilkins’s behaviour (sharing confidential data with a member of a competing team without Franklin’s explicit consent) was wrong, while Judson (1979) even claims that Wilkins stole the photograph from Franklin’s drawer. But the evidence is multi-faceted, if not downright confusing. According to Wilkins, quoted above, Gosling made it seem as if Franklin acquiesced with the transfer, but he should perhaps have asked her directly (had they still been on speaking terms). Wilkins, moreover, defends his course of action by arguing that a basic willingness to communicate and share results is a key component of the ethos of science, and that Franklin had been too possessive, in addition to the fact that it was Gosling himself who had given it to him in the first place, as Franklin was about to leave. In fact, according to Gann and Witkowski (2012, p. xi), Gosling actually was the one who had taken the crucial picture. According to Wilkins, Franklin’s male colleagues had been wrongfully “demonised” by authors for whom she emerged as a scientific martyr, an “icon of the newly prominent and vocal feminist movement” (Wilkins 2003/2005, p. 256). And the most prominent “demon” was Wilkins himself. Indeed, “the Franklin/Wilkins story has often been told as an example of the unjustness of male scientists towards their women colleagues and questions have been raised over whether credit was distributed fairly when the Nobel Prize was awarded” (Wilkins 2003/2005, p. x).

It is not my intention to pass ethical judgement in this case, in terms of right or wrong (many others have already done so). Rather, I want to point out some structural similarities between the two versions of the *Third Man* scenario we are dealing with. Both in the movie/novel and in the memoirs, a crucial piece of information is missing. Both stories are about failing to see something which in retrospect seems obvious. And in both of them, crucial information is somehow connected with the intricate position occupied by an intermediary, a (wavering and hesitant) ‘third’ man.

To analyse these similarities, let me first of all point out that the story of the ‘purloined photograph’ is reminiscent in many ways of a famous detective story by Edgar Allan Poe entitled *The purloined letter*, featuring detective Auguste Dupin. This story, which has been meticulously analysed by Lacan (1966; Muller and Richardson 1988), concerns a confidential piece of writing, an embarrassing letter—*lettre embarrassante* (Lacan 1966, p. 13)—to a Queen. The letter has been taken from her boudoir by a Minister (occupying a third, intermediary position between Queen and King) and now seems about to be handed over to the latter. In other words, it is a story about a valuable but highly sensitive piece of information that has been ‘displaced’ and is now about to embark on a complicated itinerary. As soon as it begins to circulate, it runs the risk of falling into the wrong hands (or the ‘right’ hands, depending on one’s perspective), so that it may significantly empower its new owner, although it is not evidently clear whether it will be possible (in the sense of morally admissible) for the recipient to actually *use* the information. The Queen *knows* that her letter has been taken away from her, but finds the content too embarrassing to do something about it (as this would draw too much attention to it), while the thief is subsequently faced with a similar dilemma: by using the letter, he draws attention to his misdemeanour, so that the impact of his disclosure could be thwarted. Once again, the story is about seeing and failing to see, and about having valuable information at one’s disposal, but being unable to use it.

Similar dilemmas are at work in the story of the purloined picture. Initially, Franklin seems to be the one who misses something, who fails to see. In retrospect, her perfectly X-shaped *Photograph 51* points unequivocally in the direction of a double helix structure, but for some reason she refused to accept this. She keeps the evidence hidden in her drawer, thereby repressing an inconvenient truth. For indeed, precisely because of its superb quality, the photograph (should she have decided to disclose it) would have undermined her anti-helical stance. Therefore, she apparently treats it with negligence, keeping it somewhere in her office. When she is about to leave the department, the picture suddenly shows up: i.e., the return of the repressed. It is clear that Franklin could not really *use* the picture. Her power over others depended on the fact that her valuable information, exemplified by *Photograph 51* (as a ‘condensation’ of her exceptional dexterity and expertise), remained *un*used. Franklin’s intimidating prestige depended on the fact that others (Watson, Crick, Wilkins) *knew* that she *knew* something which they did not *know*, and that she *had* something (a piece of evidence) at her disposal which they lacked. As soon as her ‘perfect’ picture, her vital piece of evidence is put into circulation, her power decreases. As soon as her trump card is brought out in the open,—because Wilkins allows Watson to see through Franklin’s cards—, her truth = power game is essentially over.

But also Wilkins himself is someone who fails to see, who misses something. While struggling with the DNA data for months, even years, something continues to escape him. How many strands are there: is it a single helix, a double or a triple one? Wilkins confesses that, for quite some time, he is in a “mental logjam” (p. 176); he cannot make up his mind. He persistently seems to follow the wrong track, notably because he and Franklin are on “different wavelengths” (p. 201), hoping for the other to leave the department, or even the field, and therefore failing to notice something (the double helical structure) which, in retrospect, seems “obvious” (p. 220). The fact that Franklin withholds her evidence paralyses Wilkins, and causes him to suffer from a “mental block” (p. 199). Because they keep “circling” around each other, as Wilkins phrases it, the Cambridge team can overtake them. But as soon as the crucial information (the missing link, in the form of an X-ray photograph) unexpectedly falls into Wilkins’s hand (on ‘neutral grounds’: the corridor at King’s), he shows it to someone else (in that same corridor), who knows how to make use of it, discerns the truth and fits the pieces together,^14^ although in retrospect the question has been raised whether the manner in which this crucial input was acquired was ethically admissible.

In essence, the DNA story is a tale of two teams, as we have seen: the King’s team and the Cambridge team. At Cambridge, teamwork clearly is a strength. As Wilkins phrases it, Watson (Jim) and Crick (Francis) “formed a powerful team” (p. 163). Because they combined complementary types of expertise (physics and biology, advanced mathematics and model-building, X-ray diffraction and phage genetics) they “formed a very effective complementary pair for the study of DNA structure” (p. 163).^15^ In the case of King’s, however, lack of collaboration was clearly a weakness. Strong coalitions, such as between Randall and Franklin on the one hand and between Franklin and Gosling on the other, were continuously pressing Wilkins into an embarrassing ‘third’ position (in terms of laboratory politics). At the time of her appointment, lab director Randall had written Franklin a confidential letter suggesting that Wilkins would leave the field of DNA crystallography to her, but Wilkins (who was not aware of the existence of this ‘secret’ letter, and only read it by coincidence many years later) had no such intentions. Time and again, he ended up being the ‘third’ man. And although he did manage to team up with ‘pro-helix’ partner Stokes for some time, the one perfect complementary combination, the duo Wilkins-Franklin, failed to match. Their relationship abounded in misunderstandings, mutual irritations and embarrassments.

A symptomatic event is the following. Wilkins, in a final desperate effort to overcome the stalemate, and prompted by the Jungian psychotherapist he was seeing, decides to invite Franklin to dinner:

I went to see Rosalind on a very warm afternoon, and found her in a large lab where she was busy fitting together the electrical wiring for the Ehrenburg fine-focus X-ray tube. She was sitting on the floor in a lab-coat and seemed quite willing to talk. The work must have been hard, for she was sweating in the heat, but she did not seem to mind the very close atmosphere in the lab. In those days before deodorants we were all used to smelling rather bad after some physical exertion, but in the stifling lab I found myself quite unable to imagine sitting down to dinner with Rosalind that day… I could no longer face the challenge of a sociable evening with her (150/1)

But the most dramatic (even tragic) dénouement of the story is the famous letter written by Wilkins to Crick on March 7 1953, shortly after the completion of the model (on 28 February 1953), in which he announces that “our dark lady” (Rosalind Franklin) will leave next week, so that Wilkins now finally feels free to start his “offensive on Nature’s secret stronghold” (i.e., the DNA structure) using all available means, including model building: “All hands to the pumps!” (Watson et al. 2012, p. 218). The phrase ‘our dark lady’ refers to Shakespeare’s sonnets, but Wilkins himself can perhaps be seen as DNA’s Hamlet: a tormented, hesitating intellectual, comparable to the Danish Renaissance prince-astronomer who mounted the platform to watch a supernova (Olson et al. 1998), but encountered a paralysing voice from the past instead. Like Wilkins, we see him time and again suspending the decisive turn, postponing the act, waiting for the optimal moment, which he allows others to determine (Lacan 2013, p. 375). And when he finally makes up his mind (“The readiness is all”), after countless detours and embarrassments, it is too late. Wilkins did receive his Nobel Prize, by way of happy end, but a most awkward situation would once again have arisen had Franklin still been alive in 1962 [since according to a long-standing rule, a Nobel Prize can be shared by three individuals at most; cf. Watson et al. (2012, p. 243)].

While relationships at King’s became increasingly tense, Wilkins’s relationship with the Cambridge team remained cordial, notably with Crick. They kept track of each other’s doings and exchanged scientific views, as well as laboratory gossip. For the Cambridge team, Wilkins functioned as intermediary; a point of contact, a kind of ‘double agent’ as it were, alternating between both teams, also on the conceptual level: Wilkins seemed constantly in doubt, desperately seeking for a ‘third’ or middle position: is it a double helix (as Jim and Francis kept insisting) or not (as the sceptics at King’s maintained, notably Franklin, who at a certain point even composed her famous obituary, declaring the double helix dead). Wilkins is a ‘messenger’, moving information back and forth, acting as the ‘third’ man, eavesdropping in both directions, and feeling increasingly uncomfortable with the situation.

Similar dynamics are at work in the movie. The story about the fatal car accident becomes increasingly unconvincing and inconsistent, but for quite some time the (apparently obvious) truth continues to escape everybody involved, until they suddenly realise that, due to their inattentiveness, their ‘tunnel vision’, they failed to look in the right place. As suspicions increase, they finally decide to open Lime’s frozen grave, and discover that he is *not* inside his coffin. He must still be moving about, continuing his game of clever escapes. As Greene phrases it, Harry had a talent for keeping track of everybody else while not being seen himself (1950/1977, p. 51). He looks at the world from a different perspective: either from high above (as in the Ferris wheel scene, discussed below) or from deep below (the sewage netherworld). He only comes into view quite suddenly, revealed by limelight (perhaps an explanation of his name): as a voyeur with a sinister smile who enjoys to be seen, but then quickly disappears again.^16^ But finally, the pieces come together, the villain can be captured, and the truth can be revealed,—but this does not imply that order is restored, as the movie’s take home message is not a reassuring happy end.

Moreover, the movie is likewise structured as a competition between two teams, searching (or hiding) valuable clues: on the one hand the pack of racketeers, on the other hand the military police. The police decided to discontinue their hunt for Harry, and the racketeers insist that he is no longer with them. Yet, there is a witness who claims that, instead of two individuals carrying off Lime’s body, right after the fatal accident, a third man had been present, someone without a face: only the top of a head had been spotted from the window (p. 67). But the racketeers insist that there was *no* third man. Martins’ presence in Vienna is an embarrassment to both teams. Both teams had expected that he would leave the city at once, and both teams consistently urge him to do so, for he constantly seems to stand in everybody’s way. As long as Martins is around, both the racketeers and the police seem inhibited to act. Gradually, Martins (who initially believed the swindlers) begins to change his mind: there must indeed have been a third man. The racketeers then decide to murder the witness, who has become a source of embarrassment to them.

And then, one night in ghostly Vienna, Martins (retreating from yet another failed attempt to seduce Anna) suddenly discerns, just around a corner, pressed against a wall to escape notice, a stocky figure. A sudden beam of light exposes him (like a phantasmagoria, produced by a magic lantern), revealing the features of Harry Lime (p. 88), the “undead” third man (Dern 2005), who apparently still keeps an eye on what is happening in his girlfriend’s apartment. He makes his escape through the labyrinthine sewer system, but due to this unexpected and revelatory event (which plays a similar role as the corridor scene in the DNA story), the mystery can now be unravelled.

At the same time, it is Martins rather than Lime who occupies the position of the ‘third man’, moving back and forth between the two teams (very much like Wilkins), picking up (apparently contradictory) pieces of information here and there, trying hard to combine them into a convincing picture so that he can finally make up his mind which side to choose. The former accomplice becomes a decoy, and in the end he even shoots his boyhood hero, after a chase through the sewer system (a chaotic acoustic Underworld of echoing voices) for which the movie is still famous.

Like in the case of Wilkins, moreover, an embarrassing relationship with a lady is involved. Martins falls in love with Anna Schmidt, Harry’s girlfriend, who, with her mysterious dark eyes, plays a role similar to that of Franklin, the “Dark Lady of DNA” (Maddox 2002), with her “watchful, dark eyes” (Wilkins 2003/2005, p. 130), nicknamed “our Dark Lady” (2003/2005, p. 210). Besides trying to solve a puzzle (the puzzle of Harry’s death for Martins, the puzzle of DNA for Wilkins), both males are in search of a female partner. “I was trying hard to find someone to marry”, Wilkins tells us (p. 120). As for Franklin, her “air of cool superiority” (p. 155) undermined Wilkins’s self-confidence from the very beginning and this, in combination with his own “juvenile attitude towards women” (p. 133), ruled out “any romantic interest in Rosalind” (p. 133). Martins is likewise depicted as a bachelor who stumbles from one erotic failure into another (referred to as “incidents”, Greene 1950/1977, p. 22), and he obviously fails to impress Anna, who is still mesmerised by Harry, her magnetic *homme fatal*. Thus, Maurice’s and Holly’s quest for truth (their ‘cupido sciendi’) becomes mixed up with their desperate yearning for intimacy. Holly hopes to form an alliance with Anna, to unravel the truth, but also in the erotic sense: he wants her to regard him as a replacement for Harry, but for various reasons they fail to match and, due to all kinds of misunderstandings between them,^17^ Harry (about to be trapped by the police) can make his final escape into the sewers, where he meets his (very Lacanian) ‘second’ death, as Martins finally knows which side to choose.

To summarize the results so far: what do Cold War espionage, detective stories, life sciences research and psychoanalysis have in common? As modes of enquiry, they all revolve around a crucial piece of information which is already there, but which is withheld, missed, overlooked or lost (repressed),—Franklin keeping the picture in her drawer, refusing to recognise the helical structure, Watson failing to take proper notes during her lecture, etc.—while others consistently fail to look for it in the right place (usually quite nearby). What key players notably fail to see is that they themselves are causing the deadlock: their own blind spots and fatal flaws are blocking the access to truth. This already applied to the case of Oedipus, of course, which may be read as an ancient Greek detective story about a crucial piece of the puzzle which, although available, is kept back, resulting in a stalemate. Information is power, however, and as soon as the missing item is revealed, the power balance shifts. Etymologically speaking, the word ‘symbol’ refers to two pieces of a broken entity (a jar, for instance), one of which is still available, while the other piece is put in circulation. And then, unexpectedly, for a brief moment, it becomes visible (Watson catching a glimpse of Photograph 51, Martins catching a glimpse of Lime in the doorway, etc.), so that the ‘symbols’ (the two complementary pieces of information) can be glued together, allowing the protagonists to recover from their impotence. In other words, Wilkins has to face the embarrassing fact that he himself is the one who is blocking the way. Unlike alter ego Martins, who becomes an alcoholic, Wilkins opts for psychotherapy, Jungian style; learning that, in order to really discern the truth, science needs a complement, namely: art.

## From soulless cities to sublimation

Harry Lime’s Vienna is a divided city, a lifeless, destitute place, broken into sectors and inhabited by trapped, ‘divided’ selves. Young men are absent (fallen in battle, arrested by the Nazis or made prisoner of war), women fend for themselves and racketeers, foreign soldiers and spies dominate the city centre. Much of the pre-war cultural ambiance (Wittgenstein’s Vienna) has likewise vanished. A skeleton wasteland remains, shot from tilted angles with canted cameras, gloomy and dark, even during daytime. Critics applauded the way the movie evoked the “spirit of place” (Gomez 1974, p. 333).

One of the cultural ‘layers’ that disappeared from this uncanny metropolis (*unheimlich* in every way) is psychoanalysis. Freud died in London while Freudians went into exile and left the Continent. Not only their physical absence is noticeable, their ‘spirit’ vanished as well. But psychotherapy, considerably weakened in Vienna, ‘triumphed’ in the United Kingdom and the United States.

Wilkins was in psychotherapy for many years. His symptoms were occasional depressions (giving rise to suicidal thoughts) and persistent difficulties with women. His first wife (Ruth) unexpectedly divorced him after only a few months of marriage.^18^ Initially, he opted for Freudian analysis, but this soon ended in failure:

I had long been interested in Freud and, after my experiences with Ruth it seemed that Freudian analysis might be useful to me. After a year of daily 8 a.m. visits to a Freudian woman therapist, arranged for me by the official Freudian organisation, I was thrown out because I reported thinking (I thought in accordance to the Freudian rules) ‘that women will never get anything out of me’ … I was in very low spirits. … I felt a bit suicidal … After the Freudian debacle, I did try psychoanalysis again. I went for advice to Dr. Bannister in Cambridge and he recommended a Jungian who specialised in marriage breakdowns. Having learnt about the unconscious from Freud, I was now interested in Jung’s ideas about thinking and feeling, and I found a very helpful analyst whom I was to visit for many years … (Wilkins 2003/2005, pp. 112–113)

Partly as a result of the Jungian approach, which fitted him much better, his interest in the connections between science and art increased.

In Birmingham he befriended two arts students who experimented with luminescent crystals similar to the ones he himself used in his research. On a self-made drawing (reproduced in the book) from this period, we see him at work late at night in his lab, while strange ideas, shaped like grotesque animals, float about: an oneiric self-portrayal in Jungian style. In Berkeley he took art classes, finding painting and drawing satisfying ways to relax after a day in the lab (p. 111). Back in London, he became attracted by (the work of) an emigrant painter from Vienna named Anna (p. 111), but their affair soon stranded. Still, art emerges as a complementary activity helping him to ‘suture’ (to use the Lacanian phrase) his chronically divided Self.

During his subsequent travels to South America he discovers new possibilities for “fusion” of science and art (p. 190). He is hugely impressed by Peruvian art, with its striking contrasts of violence and beauty. Both dimensions seem to evoke one another. Considering how the Spanish invaders smashed Inca civilisation (with its surreal “Magritte cities”) like invaders from another planet, Wilkins envisions the possible end of *all* civilisation. With profound embarrassment he ‘confesses’ to other travellers his involvement in the bomb project. These “powerful experiences” (the beauty and brutal destruction of Inca civilisation, p. 194) make him question the value of science, producing captivating truths as well as bombs.

At Cuzco, the Inca capital, he marvels at the stupendous beauty and size of the granite blocks, the mathematical skills and respect for the material needed to cut them so exactly (p. 192) reminding him of the way in which modern scientists interact with their research object. The scientists’ will to know (*cupido sciendi*) is triggered by *love* for their research object, to which scientists often become very strongly attached: “what the poet Coleridge says is still true: the scientist *loves* the material on which he or she works” (p. 156). This notably applies to his own object of choice, DNA, enigmatic, elusive and sublime:

I had been looking forward very much to being with DNA again. To use Coleridge’s expression: I *loved* DNA: I wanted to savour its nature and find what that nature revealed (p. 208).

Resisting laboratory politics, Wilkins refuses to give up DNA and designs special cameras to increase the proximity of his gaze. As one of the first visitors to see the Cambridge model, he senses that “though only bits of wire on a lab bench, [it] had a special life of its own [and seemed to speak] for itself” (p. 212).

In 1969 Wilkins became Founding President of the *British Society for Social Responsibility in Science* (BSSRS) and joined a committee on Science and Art to study the congruence between the symmetries of bio-molecular structures—studied by biophysicists with electron microscopes and X-rays (p. 254)—and the geodesic domes of architect Buckminster Fuller (Marks 1960), suggesting close convergences between molecular biology and modern art. For Wilkins, this might pave the way to broader, more interdisciplinary approaches to problems of science and society. Thus, the final chapters of his memoirs amount to a reconciliation (sublation, ‘Aufhebung’) of the science—art divide.

Again, a basic congruence with the film can be pointed out. At first glance, no two individuals seem as unlike as Wilkins and Lime. Unlike the latter, who turned deceit into a profession, Wilkins portrays himself as a conscientious researcher. And unlike Lime, the archetypal irresponsible cynic, Wilkins’s biography conveys a life-long commitment to societal issues (such as preventing nuclear warfare). At the same time, both men articulate the view that good and evil, art and violence, truth and warfare somehow belong together.

In the movie, this idea is evoked during the famous Prater Ferris wheel scene, featuring Lime as a Zarathustra-like character (Gomez 1974, p. 339), inviting Martins to follow him to an elevated position at the top of the Wheel—like Satan tempting Christ (Brown 2014, p. 29)—, from where human beings are reduced to insignificant, dispensable dots, that can easily be sacrificed without much further thought,^19^ thus elucidating his “high-spirited immorality” (Palmer and Riley 1980, p. 18), beyond good and evil. This is counterpointed, however, by the movie’s final scene when, after Lime is buried for the second time, Anna Schmidt walks towards the gate, where Holly Martins and cameraman Hans Schneeberger (former collaborator and lover of Leni Riefenstahl) await her. She starts as a Lime-like dot in the distance but, as she comes closer, she ‘individuates’ with every step, a process which culminates in her final gesture: emphatically ignoring Martins.

Welles’s impromptu rejoinder at the foot of the wheel, on the affinity between brutality and sublimity, also became quite famous:

In Italy for 30 years under the Borgias they had warfare, terror, murder, and bloodshed, but they produced Michelangelo, Leonardo da Vinci, and the Renaissance. In Switzerland they had brotherly love—they had 500 years of democracy and peace, and what did that produce? The cuckoo clock?

Although the factual correctness of this quotation is questionable of course (Switzerland excelled in research fields such as linguistics, mathematics and pharmaceutics for example, besides producing painters, authors—such as Bodmer, Keller, Meyer, Burckhardt, etc.—and the like) the basic tonality concurs with Wilkins’s memoirs, where a profound mutual entanglement is likewise discerned between warfare (violence, destruction) on the one hand and science and art on the other. Scientific breakthroughs are not only producers but also *products* of wartime activity, which explains how the scientific career of a campaigner for peace and disarmament such as Wilkins became so intimately tied up with war efforts,—from laser technology and luminescent crystals via the Manhattan project up to DNA research. Also for the latter, the connection with warfare has been clearly documented. Post-war genetics and genomics were militarised research fields from the very outset. As Cook-Deegan (1994/1995), Kay (2000) and others have shown, genomics began with interest in the impact of nuclear radiation on human DNA (which is why the U.S. Department of Energy was such an avid funder of the Human Genome Project).

As for the Great Wheel itself, a final correspondence between the cinematic and the scientific version of *The Third Man* can be pointed out. The Prater Wheel is the key symbol of the movie (Drazin 1999), the décor of the dialogue on warfare, science and life discussed above. In April 1945, the Prater Park had been the site of the ruthless final combat between the advancing Red Army and the last of the SS defenders. It was completely demolished, ‘emptied’ as it were. Only the Great Wheel (the city’s core skeleton) remained in place, symbolising a stripped-down world. But the wheel is also an (archetypal) symbol of life: the iconic cycle of growth, destruction and rebirth, associated with the Hindu deity Shiva, the cosmic dancer, annihilating a weary universe to prepare the ground for new creations. Something similar applies to the double helix. It is the core structure of the living, that which remains in place (comes into view) when the flesh of life has been removed; the starting point of renewal, connecting past and future. And as the Great Wheel (*Riesenrad* in German) is the key archetypal symbol of the movie, the double helix is the basic symbol of Wilkins’s memoirs, the thread between technologies of destruction (the Manhattan Project) and DNA research during the post-war years, the quintessential symbol of the science of *life*.

## Conclusion

What lessons can be drawn from this comparative analysis of a science autobiography and a cinema classic for understanding post-war life science research? To address this question, I will first of all unravel it into its four components:

- The *object* of analysis: post-war molecular life sciences research, culminating in the discovery of the structure of DNA (in 1953)
- The *aim* of the analysis: understanding science in retrospect (from the point of view of the present, 2015), by focussing on the micro-level of scientific research practices
- *Document 1*: a science autobiography (published in 2003)
- *Document 2*: a movie, coming from a flanking realm of culture (cinema), released in 1949

Rather than studying the scientific materials directly [such as the *Nature* publications by Watson and Crick (1953), Wilkins et al. (1953) and Franklin and Gosling (1953)], access is provided by two detours which (in cinematic terms) can be referred to as the *flashback* perspective (the science autobiography, published in 2003) and the *oblique* perspective [the cinematic classic (1949), a document coming from a flanking realm of culture, providing a sideways view]. These four components can be represented with the help of the following matrix:

**Table:** 

The question now is: what is the added value of these detours, especially when used in combination, for understanding science? In what way and to what extent do they add depth, precision and ‘resolution’ to ongoing philosophical assessments and debates?

As to the flash-back perspective, I would first of all like to refer (by way of back-flash) to the Introduction of *Science in Action* by Bruno Latour, a representative of the “anti-biographical” stance outlined above (Shortland and Yeo 1996),^20^ entitled “Opening Pandora’s Black Box” (1987, pp. 1–17). This introduction itself begins with a flashback to Watson and Crick, struggling to define a shape for DNA “compatible with the [X-ray] picture they glimpsed in Wilkins’s office” (1987, p. 2). Subsequently, Latour points out that “such flashbacks, to use the cinematic term” (p. 2), reveal the uncertain beginnings (laden with controversy and doubt) of what is usually taken for granted (‘black-boxed’) later on, in well-established science. Once a particular concept, practice or machine functions smoothly, its inner intricacies become obfuscated. In textbook knowledge, uncertainties have been removed. The basic objective of science studies, as Latour sees, it is to re-open black-boxed frictions and tensions, made invisible by the field’s subsequent success.

A similar tendency towards black-boxing, however, can be discerned in Latour’s own book. Initially, we see *individuals* at work. Watson, Crick and Wilkins are explicitly mentioned and “placed in specific situations” (p. 15). Subsequently, however, “people start being slowly erased” (p. 15) so that we end up with decontextualized textbook truths. Ironically, these same individuals disappear in Latour’s account as well. Although he aims to ‘re-open the black-box’, individual scientists are nonetheless left out of the picture. In the French version of *Laboratory Life* (Latour and Woolgar 1996), it is explicitly stated that what individual scientists have to say about their own work must be discarded as doubtful. They are too much participants to act as a reliable observers.^21^ In other words, in the classic STS approach, the micro-level of scientific individuals (as represented in science memoirs) continues to be black-boxed and the basic objective of the biographical ‘renaissance’ heralded by Söderqvist and others is to open up this final black-box as well, as a privileged (but often eclipsed) space or node where some of the key challenges and uncertainties of laboratory life become visible, in high resolution format.

What does the movie add to such an endeavour? In a previous section it was explained, building on Söderqvist (2006, 2011), that I see science autobiographies mainly as (a) a method for writing *contextual* history and (b) an exercise in research *ethics*. On both levels, I would argue, the comparative analysis of the movie adds resolution to the insights provided by the biographical materials. In terms of context, the movie strengthens our sensitivity, not only to the socio-cultural and topological ambiance (backdrop) of the scientific drama (Lime’s post-war Vienna obliquely mirroring Wilkins’s post-war London), but also to the ambiguities and intricacies of the moral dimension, i.e., the dramatic action itself. Both in laboratories and in the outdoors world, individuals in various settings were facing challenging, and in many ways comparable dilemmas concerning the use of sensitive, valuable and confidential information about alluring, but at the same time risky scientific entities that should somehow be contained. The congruencies are *mutually* revealing, often in unexpected ways. By probing moral quandaries (concerning sensitive information, gender issues and the militarisation of science) in *different* settings, their ambiguities become more pronounced. The comparative approach reveals how these issues were intimately interwoven with broader social and cultural trends, as ‘symptoms’ of post-war culture, but at the same time they are seen through the lens of a movie which, in view of its unrelenting popularity, helped to shape our current image of the post-war era, in combination with an autobiography which aims to bring these issues back to life, but coloured by five decades of hindsight and reflection. In other words, although these documents purport to tell us something about the post-war past (the upper-left cell in the matrix), both the lasting popularity of the movie and the revival of interest in science autobiographies indicate that they also tell us something about the present, namely about the way in which post-war scientific dilemmas are framed and perceived today (the lower-right cell in the matrix).

Instead of *The Third Man*, I could have chosen the movie *Life Story*, about the discovery of DNA, based on personal accounts by Watson, Crick and others and released in 1987, beginning with “Watson’s bungled effort to enter into a dialogue with Wilkins” (during an excursion at Paestum) and ending with the compelling, awe-inspiring DNA model, sublime and immortal, “rotating to celestial music” (Crick 1988, p. 85). *Life Story* certainly adds a visualised, dramatic layer to the memoirs (a vivid enactment of the uncertainties and desires of key protagonists). At the same time, it lacks both the discursive richness of autobiographical materials (which is reduced to a limited number of concise dialogues) and the unexpected findings provided by the oblique approach (the comparison with a contemporary movie depicting a different cultural domain than science). In the case of *Life Story*, the focus of comparison would be on adequacy: does the movie represent the recorded events in a truthful manner? In short, *Life Story* concurs with a *flash back* approach, rather than with the *oblique* approach. Those familiar with the memoirs will basically know what to expect. The comparative analysis with *The Third Man* movie, however, is far more experimental.

All three co-discoverers credited with the discovery of the structure of DNA in 1962 have published memoirs (Watson 1968; Crick 1988; Wilkins 2003/2005) and in each case the title contains a reference to fiction. Watson’s book was initially entitled *Lucky Jim* (after Kingsley Amis’s 1954 novel), while Crick’s title (‘What mad pursuit’) was taken from the poem *Ode to a Grecian Urn*, written by John Keats in 1819. Would a comparative anatomy work in these two cases as well? In Watson’s case (a novel about an academic who eventually manages to get his girl) the parallel seems too obvious to be revealing. And although in the case of Crick some interesting connections can be made, for instance with DNA as music (“unheard melodies”; cf. Noble 2006) or with “Beauty is truth, truth beauty” (think of the appealing/convincing helical beauty of the model), Keats’s poem belongs to a different era. The mutual exposure (the oblique perspective) works best when a contemporary document (movie, novel, play, artwork, etc.) is used (Zwart 2008b). The movie version of *The Third Man* not only reflects the temporal (cultural, normative) ambiance of the scientific events, moreover. As a cinematic *classic*, it also effectively colours the way in which the post-war period is perceived today, so that the arrows in the matrix should actually point in both directions. In the end, the comparative analysis deepens our self-understanding, i.e., our understanding of the present as a normative ambiance for developing critical reflections on the post-war past.

## Footnotes

1 Some indications: it not only won the *Grand Prix* in Cannes in 1949 and the *British Film of the Year Award* for 1949, but still ranked second in *MovieMail* 2000, was voted best British film ever by the prestigious British Film Institute in 1999, and is in third position on the Rotten Tomatoes list of the best mystery/suspense movies. The title music (‘Third Man Theme’), which topped the international music charts in 1950, has likewise remained a well-known popular tune (http://en.wikipedia.org/wiki/The_Third_Man).

2 In the days of the discovery of the DNA structure, *The Third Man* was a very famous movie, and Orson Welles a very famous actor. Although *The Third Man* is not explicitly discussed by Wilkins, he does refer to another much-acclaimed movie starring (as well as being co-written, directed and produced by) Orson Welles, namely *Citizen Kane*, comparing the managerial style of his boss John Randall to that of Charles Forster Kane: “In the College at Christmas… Randall’s party was like a scene from Orson Welles’ *Citizen Kane* where Kane celebrates the success of his great newspaper by joyously leading his dancing staff across the stage” (p. 102).

3 The societal responsibilities of scientists were very much on Wilkins’s mind while writing his memoirs: “The later years of my career have been devoted to the exploration of the social issues raised by advances in science” (Wilkins 2003/2005, p. x). He played a prominent role in the post-war *British Society for Social Responsibility in Science* (BSSRS), campaigning for nuclear disarmament.

4 The post-war atmosphere, the sense that we are heading towards self-imposed destruction is ubiquitous in the movie, which has a great city in shambles as its gloomy backdrop, but this same atmosphere is also present in Wilkins’s book, for instance when he reports his experiences upon visiting the remains of Inca civilisation and its “surreal ruins of brutal destruction” (p. 194), suggesting the possible end of *all* civilisation and evoking in him a strange feeling of “detachment” (discussed in the final section of this paper).

5 “War-ravaged Vienna [was] so radically disrupted that it ceased to be a functioning ethical community” (Brown 2014, p. 36). The black market was omnipresent, cigarettes were currency and everything was commoditized, including passports, sex and pills.

6 “The film does not grapple with Nazism, nor does it take sides in the East–West confrontation” (Schwab 2000, p. 3); “We had no desire to move political emotions” (Green 1950/1977, p. 11).

7 The experience of DNA as something which speaks out, addressing us, is conveyed by Watson in the movie *Life Story* (released in 1987) when he exclaims, after having been shown photograph 51: “I could not believe my eyes; it was just sitting there, yelling out information, like a speak your weight machine” (29:20).

8 In January 1950, he was sentenced to fourteen years of imprisonment. After his release (in 1959) he emigrated to the German Democratic Republic, was elected to the Academy of Sciences and became director of the Institute for Nuclear Research in Rossendorf.

9 “Wilkins had been investigated by MI5 and the FBI. They suspected that one of nine scientists from New Zealand or Australia had leaked A-bomb secrets. Wilkins was one of the nine suspects, having worked on the Manhattan project” (Gann and Witkowski 2012, p. 123). His mail was searched and his phone was tapped.

10 The first James Bond movie was released in 1962, the year in which Watson, Crick and Wilkins received their Nobel Prize.

11 Drazin (1999) even sees *The Third Man* as a “coded message” coming from Greene, directed at Philby, who “could have read in it his past, his present and his future” (p. 151).

12 Palmer and Riley (1980) argue that Martins represents the prototypical American: the author of Westerns whose Western-like style of handling situations seems inadequate and out of place in post-War Vienna. Insisting on moral unambiguity, he remains insensitive to complexities of post-War Europe. Lime represents the ‘other’ American, more at home in the moral vacuum of a divided, ravaged city: the unscrupulous entrepreneur.

13 The amounts of adenine (A) and thymine (T) as well as the amounts of guanine (G) and cytosine (C) in DNA are approximately equal, suggesting a symmetrical structure for the DNA molecule.

14 After Watson had taken in photograph 51, two other pieces of the puzzle were still missing. On the one hand the hint, coming from crystallographer Jerry Donohue, that tautomeric representations of nucleotides reflected a keto rather than an enol configuration (suggested in the manuals he consulted); on the other hand crucial information on the C2 space group, mentioned in a confidential MRC assessment report and given to Watson and Crick by the latter’s roommate Max Perutz, member of the committee (Perutz was severely criticised for this handling of sensitive information: Watson, Gann and Witkowski 2012, p. 195). Before these final pieces became available, Watson had tried the movies, hoping that cinematic experiences would provide him with some intuitions: “I went ahead spending most evenings at the films, vaguely dreaming that any moment the answer would surely hit me. Occasionally my wild pursuit of the celluloid backfired, the worst occasion being an evening set aside for *Ecstasy*… Even during good films I found it impossible to forget the bases. The fact that we had at last produced a stereochemically reasonable configuration for the backbone was always in the back of my head” (Watson, Gann and Witkowski 2012, p. 193/4).

15 The same can be said about the Reed/Greene team: “Carol Reed and I worked closely together… No third ever joined our conferences” (Greene 1950/1977, p. 10).

16 He is both sub*lime* (elevated, looking at a minuscule world below) and subliminal (hidden from view, making his appearance extremely late: sixty-five minutes into the film, emerging from the heart of the darkness.

17 Franklin was quite unimpressed by Wilkins, and the same goes for Anna Smith where Holly Martins was concerned. In the movie, Anna says, when Holly tries to win her over: “If you’d rang me up and asked me whether you’re fair or dark or had a moustache, I wouldn’t have known”. Moreover, she keeps calling him ‘Harry’, a tenacious Freudian slip. In the novel, she states: “I don’t want him [Harry], but he’s in me… When I have a sex dream, he’s always the man” (Greene 1950/1977, p.109).

18 “After a few months [Ruth told me] that she had made an appointment for me with a lawyer, and when I arrived at his office I was shocked to hear from him that Ruth wanted to end our marriage” (Wilkins 2003/2005, p. 86). Their relational breakdown virtually coincided with the dropping of the atomic bombs, in August 1945.

19 With the possible exception of Anna, for, although he betrays her to the Russians (who are bent on arresting her because, as the daughter of a Nazi father, she is living on forged papers), he almost unnoticeably draws an arrow-shot heart with Anna’s name on the misted glass of the cabin window, before more or less handing her over to Holly (Brown 2014, p. 28).

20 His “The Pasteurisation of France” (Latour 1984/1988) has been referred to for instance as a *Hamlet* without Hamlet, since the hero whose name is referred to in the title is virtually absent in the book (Shortland and Yeo 1996).

21 « Pour donner un peu d’indépendance aux analyses de la science, il est donc nécessaire de ne pas se reposer uniquement sur ce que les savants et chercheurs disent d’eux-mêmes. Ils doivent devenir ce que l’ethnologie nomme un « informateur » , un informateur certes privilégié, mais enfin un informateur dont on doute » (Latour and Woolgar 1996, p. 17; cited in Graham 2004, p. 57).
